# Social Contact Patterns in Rural and Urban Settings, Mozambique, 2021–2022

**DOI:** 10.3201/eid3101.240875

**Published:** 2025-01

**Authors:** Moses C. Kiti, Charfudin Sacoor, Obianuju G. Aguolu, Alana Zelaya, Holin Chen, Sara S. Kim, Nilzio Cavele, Edgar Jamisse, Corssino Tchavana, Americo Jose, Ivalda Macicame, Orvalho Joaquim, Noureen Ahmed, Carol Y. Liu, Inci Yildirim, Kristin Nelson, Samuel M. Jenness, Herberth Maldonado, Momin Kazi, Rajan Srinivasan, Venkata R. Mohan, Alessia Melegaro, Fauzia Malik, Azucena Bardaji, Saad B. Omer, Ben Lopman

**Affiliations:** Emory University, Atlanta, Georgia, USA (M.C. Kiti, A. Zelaya, H. Chen, S.S. Kim, S.M. Jenness, C.Y. Liu, K. Nelson, B. Lopman); Manhica Health Research Center, Manhica, Mozambique (C. Sacoor, E. Jamisse, C. Tchavana, O. Joaquim); Ohio State University, Columbus, Ohio, USA (O.G. Aguolu); Yale University, New Haven, Connecticut, USA (O.G. Aguolu, I. Yilirim); Polana Canico Health and Research Centre, Maputo, Mozambique (N. Cavele, A. Jose, I. Macicame); University of Texas Southwestern Medical Center, Dallas, Texas, USA (N. Ahmed, F. Malik, S.B. Omer); Universidad del Valle de Guatemala, Guatemala City, Guatemala (H. Maldonado); Aga Khan University, Karachi, Pakistan (M. Kazi); Christian Medical College–Vellore, Vellore, India (R. Srinivasan, V.R. Mohan); Bocconi University, Milano, Italy (A. Melegaro); ISGlobal–Barcelona Institute for Global Health, Barcelona, Spain (A. Bardají)

**Keywords:** COVID-19, SARS-CoV-2, coronavirus disease, severe acute respiratory syndrome 2, viruses, ocial contact patterns, rural populations, mathematical modeling, vaccines, Mozambique

## Abstract

Few sources have reported empirical social contact data from resource-poor settings. To address this shortfall, we recruited 1,363 participants from rural and urban areas of Mozambique during the COVID-19 pandemic, determining age, sex, and relation to the contact for each person. Participants reported a mean of 8.3 (95% CI 8.0–8.6) contacts per person. The mean contact rates were higher in the rural site compared with the urban site (9.8 vs 6.8; p<0.01). Using mathematical models, we noted higher vaccine effects in the rural site when comparing empirical (32%) with synthetic (29%) contact matrices and lower corresponding vaccine effects in the urban site (32% vs 35%). Those effects were prominent in younger (0–9 years) and older (≥60 years) persons. Our work highlights the importance of empirical data, showing differences in contact rates and patterns between rural and urban sites in Mozambique and their nonnegligible effects in modeling potential effects of vaccine interventions.

Human social contact patterns drive the transmission of pathogens that spread through proximity. Data on social contact patterns are critical to understand who contacts whom and infer who acquires infection from whom, providing insight on potential control measures, such as physical distancing and vaccination. Underlying the patterns of contact are demographic, sociocultural, and economic determinants, which vary within and across regions, resulting to corresponding variation in contact patterns. Unfortunately, such critical data are not as widely available in low- and middle-income countries (LMICs), including Mozambique (*1*), as they are in high-income countries (HICs) (*2*). Existing data on social contact patterns were collected across rural-urban divides (*3*–*6*) and informal settlements (*7*,*8*), limiting the representativeness of the results. Recent studies incorporate innovative methods to obtain data from infants and illiterate persons by using shadows (*3*,*9*), interviewer-led questionnaires (*4*,*5*), or wireless proximity sensors (*10*,*11*). Simulated contact rates for LMIC populations have been derived by projecting empirical data collected from HICs (e.g., POLYMOD data) and scaling using local demographic patterns (*12*). However, those extrapolations likely mischaracterize contact patterns in important ways when they differ for reasons aside from demographics.

During the early phase of the COVID-19 pandemic, the limitations inherent to estimating human contact patterns became apparent on a global scale (*13*–*17*). In the absence of vaccines or pharmaceutical interventions, physical distancing (i.e., reducing the number and riskiness of contacts) was implemented. Relatively little data were collected from LMICs, limiting the ability of health officials to quantify any changes and employ such data in developing contextual models of intervention.

Starting in 2021, during the COVID-19 pandemic, we launched the GlobalMix Study to collect social contact data from 4 LMICs: Mozambique, Guatemala, India, and Pakistan. We collected data from selected rural and urban areas using methods that were customized for each country (*18*). Here, we present the methods and results from Mozambique, for which we have complete datasets.

## Methods

### Study Objectives

The main aim of this study was to characterize the patterns of social contact with respect to directly transmitted infections. We then simulated the transmission of a hypothetical respiratory virus and assessed the effects of vaccination in a model using contact data generated from this study (henceforth called empirical data) and compared with synthetically constructed contact data (henceforth called synthetic data).

### Study Design

We conducted our cross-sectional study during March 2021–April 2022. Our data collection period coincided with active SARS-CoV-2 transmission in Mozambique (*19*). The rural site was within the Manhiça Health and Demographic Surveillance System (*20*); the urban site was in Maputo City within the Polana-Caniço Demographic and Health Surveillance System (*21*). Before collecting the social contact data, we held 25 focus group discussions and 40 cognitive interviews with community members drawn from the 2 sites. We aimed to understand the determinants of human interaction at the study sites and explore the perceptions, acceptability, and utility of paper diaries for collecting data. We also hoped to get community buy-in and useful practice recommendations on our research implementation process.

Complete details of the sample size, data collection tools and procedures, and data analysis methods have been described in our protocol (*18*). In brief, we aimed to collect data from 630 persons per site, randomly selected by age and sex from the Demographic and Health Surveillance System registers. Participants were requested to keep a paper diary of their social contacts (henceforth called contacts) over 2 days, defined as a 2-way, face-to-face encounter that involved either physical touch (skin-to-skin touch or over clothes) or nonphysical interaction (a conversation involving >2 persons while standing within arms’ length of each other and with no physical barrier between them). Additional qualitative questions are available in the shared codebook (see Acknowledgments). Field workers captured data electronically in REDCap forms (*22*) coded in portable electronic tablets. All children <10 years of age and illiterate persons ≥10 years of age selected, or were assigned, a shadow to discretely record contacts on their behalf; this process did not require that shadows follow participants all day.

### Data Analysis

#### Characteristics of Contact Patterns

We estimated the mean (with bootstrapped 95% CIs) and median (with interquartile ranges) contact rates per person over 2 days and for day 1 only. Assuming *x_ij_* represents the total number of contacts between participants in age group *i* and contacts in age group *j*, we calculated the mean number of reported contacts (*m_ij_*) as *x_ij_*/*n_i_*, where *n_i_* was the study population in group *i*.

We stratified the mean contact rates by site (rural or urban), age, sex, day of the week (weekday or weekend), type of contact (physical or conversation only), household membership (household member or nonhousehold member), occupation (or daily activity), and whether the participant reported symptoms of acute respiratory infection (ARI) or acute gastroenteritis (AGE) within the 14 days before the survey. We used the Wilcoxon rank-sum test to assess the difference between median contact rates within the sites for each covariate as well as between the rural and urban sites.

We computed age-stratified contact matrices to quantify the interactions between age groups. We adjusted the contact matrices to account for reciprocity, assuming that the total number of contacts from age group *i* to *j* were equal to the number of contacts from age group *j* to *i*: that is, *m_i_*_,_*_j_* = *m_j_*_,_*_i_* (*6*). We presented the age-specific contact matrix using data from day 1 only by using the revised formula (*m_i_*_,_*_j_* + *m_j_*_,_*_i_*)/(*n_i_* + *n_j_*).

#### Characterizing Location-Specific Proximity Contact Exposures

We compared close contacts (those individually recorded in diaries) with proximity contacts (co-location with others but without direct interaction) on the basis of information that was collected by using a place-use survey. We described the number of unique visits at various locations (other person’s home, street, market or shop, transport hub, agricultural field, school, work, place of worship, well, playground) and the distribution of time spent at each place. We then compared the number of proximity contacts at each location to the total close contacts at the same location and examined these data for differences in patterns between the urban and rural site.

#### Vaccine Effects Modeling

**Figure 5:**
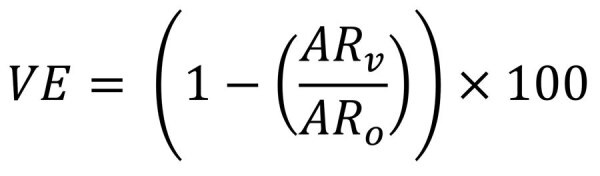
Equation. Overall vaccine effect (VE) calculated as the percent reduction of cases in the presence versus absence of vaccine.

We compared rural and urban respiratory virus transmission models parameterized with empirical data to those parameterized by using synthetic contact data (*12*). We built a deterministic susceptible-infectious-recovered model with a vaccine conferring protection against infection. We computed the mean number of contacts by reclassifying the participants into six 10-year age classes, 0–59 years, and 1 age group for persons ≥60 years of age for compatibility with the synthetic data. We weighted the empirical contact rates by using 2021 rural and urban Mozambique population distribution data and adjusted for reciprocity by using the socialmixr R package (https://github.com/epiforecasts/socialmixr). We modeled vaccination as leaky, providing partial protection for those vaccinated (50% vaccine coverage, 50% effectiveness); we modeled duration of illness as 7 days and fixed the basic reproduction number at 2.5. We calculated the attack rate for no vaccine (AR_0_) and vaccine (AR_v_) scenarios separately for rural and urban sites and presented the overall vaccine effect (VE) calculated as the percent reduction of cases in the presence versus absence of vaccine (Figure 5)

We used the EpiModel R package to run all transmission models. We conducted analyses using R v4.3.2 (The R Project for Statistical Computing, https://www.r-project.org).

## Results

### Baseline Characteristics of Participants

Out of 1,693 residents approached, we retained 1,363 participants across both sites (81% participation rate). We noted similar participation rates in the rural (676/800 [85% participation rate]) and urban (687/893 [86% participation rate]) sites. We exceeded our target sample size by 103 participants, particularly in those 40–59 years of age. Of the 1,363 participants, 666 (49%) were female and 697 (51%) male, and sex was equally distributed by site. By site, there was no major difference in number of participants recruited by age, sex, or school enrollment status (Table 1).

**Table 1:** Characteristics of study participants in rural and urban sites in Mozambique in a study of social contact patterns, 2021–2022

Characteristic	No. (%) participants		
	Overall, N = 1,363	Rural, n = 676	Urban, n = 687
Sex*			
F	666 (49)	332 (49)	334 (49)
M	696 (51)	343 (51)	353 (51)
Participant age			
<6 mo	128 (9)	62 (9)	66 (10)
6–11 mo	146 (11)	82 (12)	64 (9)
1–4 y	135 (10)	63 (9)	72 (10)
5–9 y	122 (9)	64 (9)	58 (8)
10–14 y	125 (9)	61 (8)	64 (9)
15–19 y	124 (9)	64 (9)	60 (9)
20–29 y	125 (9)	64 (9)	61 (9)
30–39 y	125 (9)	64 (9)	61 (9)
40–59 y	209 (15)	89 (13)	120 (17)
≥60 y	124 (9)	63 (9)	61 (9)
Able to read and write			
Y	701 (51)	293 (43)	408 (59)
Currently enrolled in school			
Y	368 (28)	173 (26)	195 (29)
Occupation or daily activity†			
Child	274 (23)	144 (24)	130 (22)
Unemployed	162 (14)	97 (16)	65 11)
Student	324 (27)	153 (26)	171 (29)
Homemaker	33 (3)	9 (2)	24 (4)
Casual laborer	78 (7)	22 (4)	56 (9)
Farmer	70 (6)	64 (11)	6 (1)
Businessperson	66 (6)	7 (1)	59 (10)
Office worker	83 (7)	27 (5)	56 (9)
Retired	20 (2)	5 (1)	15 (3)
Other	74 (6)	58 (10)	14 (2)
Regular mask use			
Y	942 (69)	435 (64)	507 (74)
Acute gastroenteritis: diarrhea/vomiting	27 (2)	15 (2)	12 (2)
Acute respiratory infection, >1 symptom	233 (17)	122 (18)	111 (16)
Who filled the diary?			
Self	665 (49)	361 (54)	304 (44)
Fieldworker	698 (51)	315 (46)	383 (56)

*Two participants did not report their sex. †A total of 179 participants did not report their occupation or daily activity.

The mean household size was 5.5 (range 1–18) in the rural site and 5.7 (range 1–20) in the urban site. Overall, 379 (45%) households had 4–6 members, and 4 had 1 resident. When we omitted children, students, and unemployed persons, the most common reported occupations in the urban site were business people (16%, 59/366), office workers (15%, 56/366), and casual laborers (15%, 56/366). Omitting those same cohorts, we noted 16% (64/394) of workers in the rural site were farmers. More than half (69%, 942/1,363) of participants reported wearing a mask inside or outside the house. About one fifth (233/1,363) of participants reported having >1 ARI symptom, and 26 (2%) reported >1 AGE symptom.

Half (51%, 701/1,363) of the participants were able to read and write. Most (88%, 1,200/1,363) of the participants said that they reported all contacts. However, 51% (698/1,363) required assistance from a field worker to fill out the diary at the end of the 2 days (rural 43% vs. urban 56%). Generally, all children <5 years of age (409/1,363) had a family member as a shadow; of those, 243 (50%) required additional assistance from the fieldworker. Among other ages, there was no difference in proportion of those requiring a shadow (or help from fieldworker) compared with no help, apart from age groups 15–19 years (33%, 41/124) and ≥60 years (60%, 75/124). Eight participants reported testing positive for SARS-CoV-2, all of whom reported either going to quarantine (government facility, n = 5) or self-isolating at home (n = 3).

### Contact Patterns

Participants reported a total of 17,674 contacts over 2 days; 41% were reported as unique contacts (n = 3,904 day 1 only; n = 3,250 day 2 only) and 59% (n = 10,304) were reported on both days (repeat contacts). Participants reported an overall mean of 13.1 (95% CI 12.6–13.5) contacts over 2 days (Table 2). We observed a significant difference in the mean number of contacts reported on day 1 (mean 8.3 [95% CI 8.0–8.6]) compared with day 2 (mean 5.5 [95% CI 5.3–5.7]) (p<0.01 by paired t-test). Because diary completion dates were randomly assigned, the actual mean contacts should not vary between the first and second date of diary completion. Therefore, we believe that the observed difference was a result of reporting bias that resulted from participant fatigue; henceforth, we report the mean and median number of contacts on day 1 only.

**Table 2:** Median and mean number of contacts on day 1 by demographic characteristic reported by participants in study of social contact patterns, Mozambique, 2021–2022*

Characteristic	Rural			Urban	
	Median (IQR)	Mean (bootstrapped 95% CI)		Median (IQR)	Mean (bootstrapped 95% CI)
Sex					
F	9 (6–11)	9.6 (8.9–10.2)		6 (4–8)	6.7 (6.3–7.1)
M	9 (7–13)	10.1 (9.5–10.6)		6 (4–9)	7 (6.6–7.4)
Age					
<6 mo	8 (5–10)	7.8 (6.8–8.9)		4 (3–5)	4.4 (3.8–4.9)
6–11 mo	8 (6–11)	8.6 (7.7–9.4)		5 (3–7.5)	5.8 (5–6.6)
1–4 y	9 (7–10)	8.9 (8–9.9)		6 (4–9)	7.1 (6.1–8.1)
5–9 y	9 (6.8–12)	10.1 (8.6–11.6)		6 (4.25–9)	7.2 (6.1–8.2)
10–14 y	10 (8–14)	11 (9.5–12.4)		8 (6–10.3)	8.7 (7.7–9.7)
15–19 y	12 (8.8–17)	12.8 (11.4–14.2)		9 (6–12)	9.6 (8.3–10.9)
20–29 y	9 (7–12.25)	10.8 (9.3–12.3)		6 (4–8)	6.1 (5.5–6.8)
30–39 y	8.5 (7–11)	9.4 (8.1–10.7)		6 (4–9)	7 (6.1–7.9)
40–59 y	10 (6–13)	11 (9.5–12.5)		6 (5–8.5)	6.8 (6.1–7.5)
≥60 y	6 (4–10)	7.4 (6.1–8.7)		5 (4–8)	5.6 (4.8–6.4)
Occupation or daily activity					
Child	8 (5–10)	8.2 (7.6–8.9)		4 (3–6)	5.1 (4.6–5.6)
Unemployed	7.5 (5–12)	8.9 (7.7–10)		6 (4–9)	7.1 (5.9–8.2)
Student	10 (8–14)	11.4 (10.5–12.3)		8 (5–10)	8.4 (7.7–9)
Homemaker	9 (7–10)	8 (5.2–10.8)		6 (4.75–8)	6.8 (5.5–8)
Casual laborer	8 (6–11.5)	9.5 (6.9–12.1)		5.5 (4.8–7)	6 (5.4–6.7)
Farmer	9 (6–13.25)	10.6 (8.9–12.4)		3.5 (3–4)	3.8 (2–5.6)
Businessperson	10 (7.5–11.5)	10.1 (5.1–15.2)		6 (4–8)	7.1 (6–8.3)
Office worker	8 (7–11)	9.9 (7.3–12.4)		5 (3.5–5.5)	5.1 (3.4–6.9)
Retired	5 (4–6)	5.6 (2.2–9)		10 (8.3–10)	9.1 (7.2–11.1)
Other	10 (7–13)	11.1 (9.5–12.7)		NA	NA
Household size					
1	6 (4–9.3)	7.2 (5.4–8.9)		4 (2.5–7)	5.2 (2.4–8)
2–3	7 (4–10)	8.1 (6.9–9.2)		5 (3–7.5)	5.6 (4.9–6.3)
4–6	9 (6–12)	9.6 (8.8–10.3)		6 (4–8)	6.5 (6–7)
7–10	10 (8–13)	10.9 (9.9–11.9)		7 (5–9)	7.8 (6.8–8.7)
≥10	14 (9.5–16.5)	13.8 (12–15.7)		9 (6–12)	9.6 (7.9–11.4)
Household membership					
Member	9 (6–12)	10 (9.6–10.4)		6 (4–9)	6.9 (6.6–7.2)
Nonmember	7 (4–9.5)	7.6 (6–9.2)		5 (3–8)	5.7 (4.7–6.8)
Enrolled in school					
Y	9 (7–13)	10.8 (9.9–11.7)		8 (5–10)	8 (7.4–8.6)
N	9 (6–11)	9.4 (8.9–9.9)		6 (4–8)	6.3 (6–6.7)
Weekday or weekend					
Weekday	9 (6–12)	9.8 (9.3–10.3)		6 (4–9)	7 (6.6–7.3)
Weekend	9 (6–12)	9.8 (9.1–10.5)		6 (4–8.5)	6.4 (5.9–7)
ARI symptoms (>1 symptom)	10 (7–13)	10.7 (9.7–11.6)		6 (4–8)	7.1 (6.3–7.9)
AGE symptoms	9 (8–13)	9.7 (7.2–12.1)		6 (4–8.5)	6.6 (4.5–8.8)

*AGE, acute gastroenteritis; ARI, acute respiratory infection; IQR, interquartile range; NA, not applicable.

**Figure 1:**
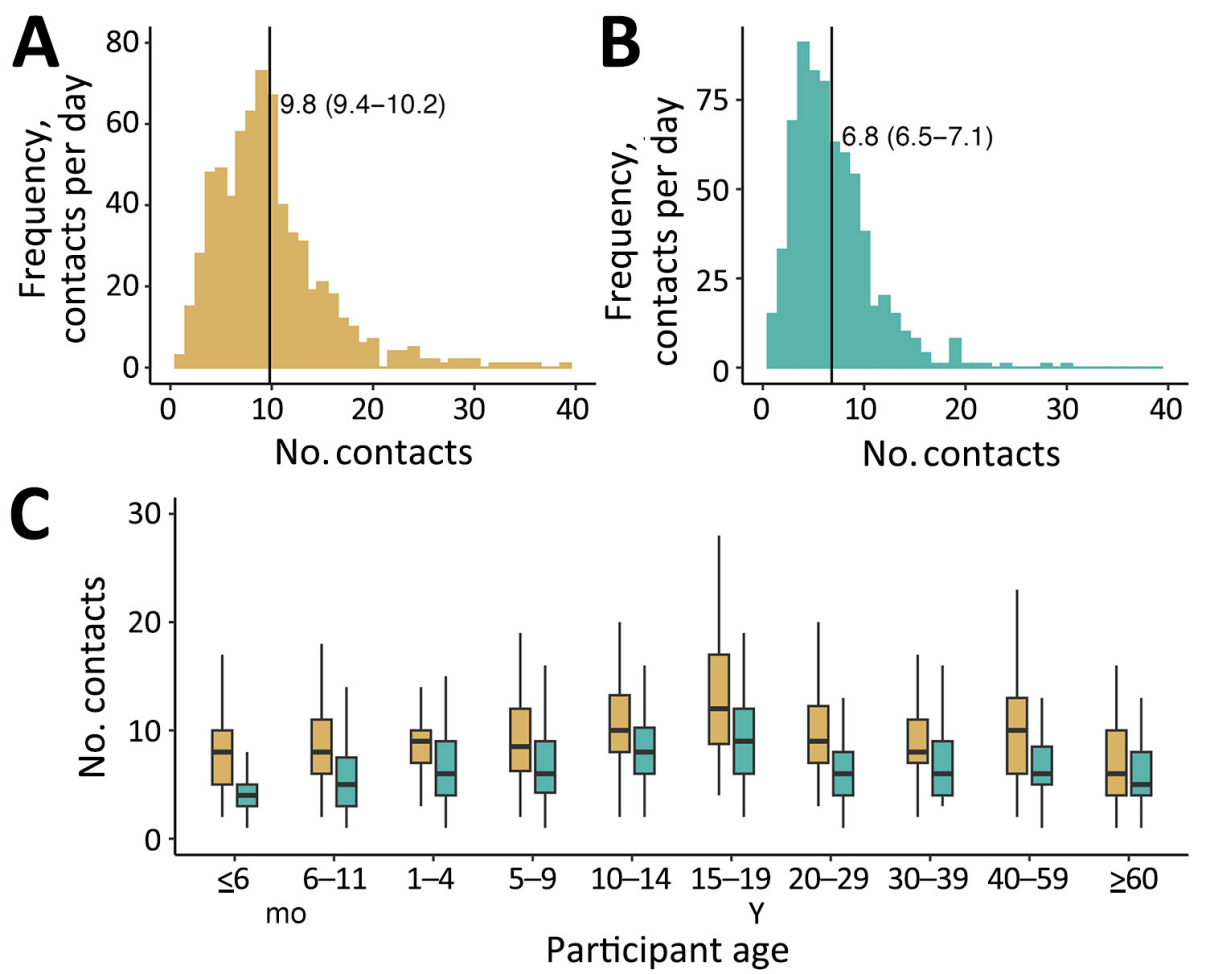
Distribution patterns of number of contacts in rural and urban areas in study of social contact patterns, Mozambique, 2022. A, B) Density distribution of the number of contacts per person in the rural (A) and urban (B) sites. Black vertical lines indicate means; 95% CIs are provided in parentheses. C) Boxplots of the distribution of number of contacts by site (gold, rural; aqua, urban). Horizontal lines within boxes indicate median number of contacts; top and bottom lines indicate interquartile ranges; error bars indicate 95% CIs.

The rural mean contact rate (mean 9.8 [95% CI 9.4–10.2]) was significantly higher than the urban rate (mean 6.8 [95% CI 6.5–7.1]) (p<0.01) (Figure 1, panels A, B). Contact rates were higher in rural areas for each age group (Figure 1, panel C). The rural mean number of contacts with nonhousehold members was significantly higher than contacts with household members (6.6 vs. 3.9; p<0.01), but there were marginal differences for participants in the urban site (4.2 vs. 3.6; p = 0.46). Corresponding median values for day 2 are provided (Appendix Table 1).

Physical contacts were, on average, more numerous than conversation-only contacts in both rural areas (6.7 vs. 4.9; p<0.01) and urban areas (5.3 vs. 3.3; p<0.01). Participants ≤18 years of age were the main drivers of the higher number of physical contacts. Of all participants, 803 (59%) reported having the same number of social contacts compared with periods before the COVID-19 pandemic. From those 803 participants, urban participants reported either significantly fewer (n = 238; mean 5.9 [95% CI 5.5–6.3]) or more (n = 28; mean 11.7 [9.3–14.1]) contacts compared with those who reported no change (n = 410; mean 7.0 [6.7–7.4]). In the rural site, 74 (11%) of participants reported more mean contacts than usual (mean 12.6 [95% CI 10.9–14.3]), different from those who reported either no change (n = 384; mean 9.6 [95% CI 9.1–10.1]) or fewer contacts (n = 215; mean 9.2 [95% CI 8.5–9.9]).

#### Contact Matrices

**Figure 2:**
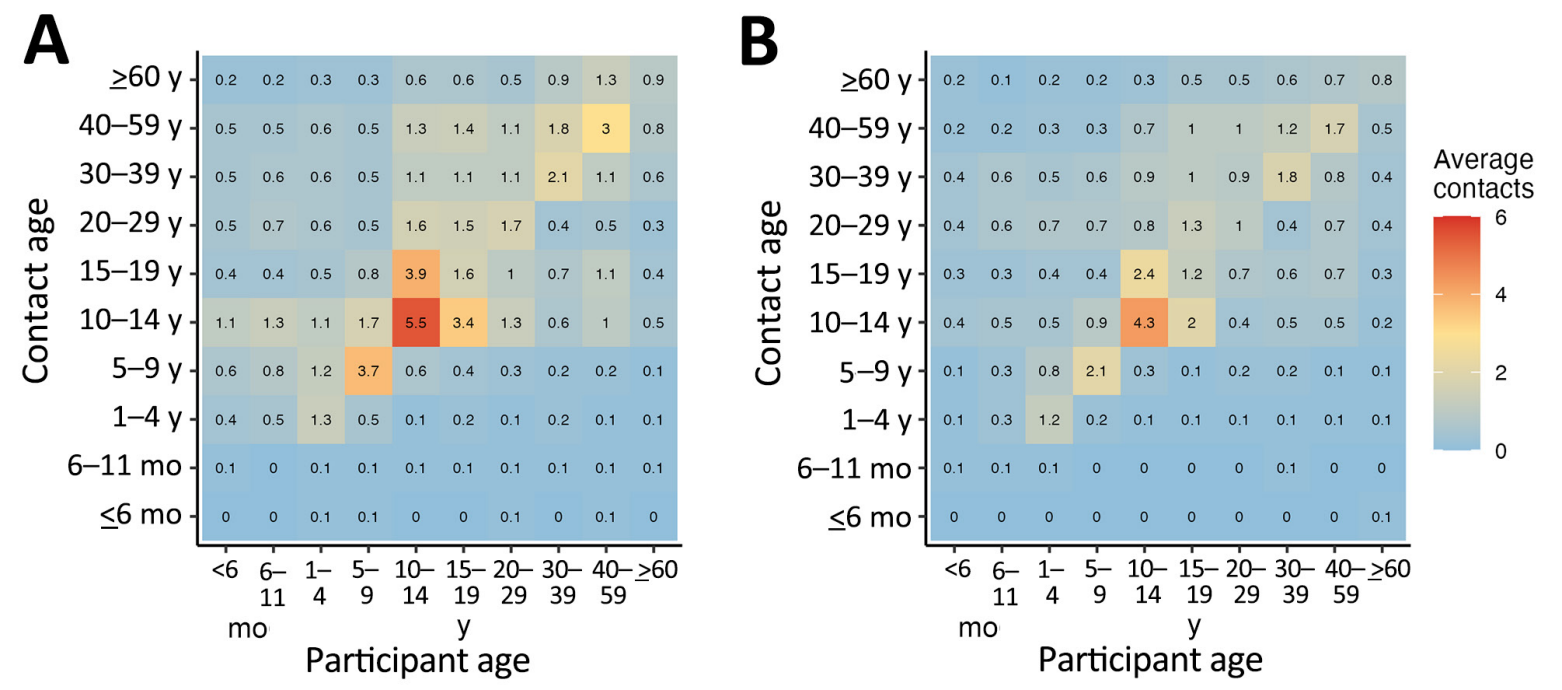
Age-specific contact matrices for rural (A) and urban (B) sites from study of human contact patterns, Mozambique, 2022. Matrices depict the average mean number of persons in age group *j* (y-axes) with whom a participant in age group *i* (x-axes) came into contact.

The urban matrix suggests lower mean number of contacts across all ages compared with the rural site (Figure 2). Participants 5–14 years of age (school-going children) and working adults 30–59 years of age in both rural and urban areas reported higher assortative (same age) mean contacts. Older school-goers 15–19 years of age reported, on average, a high number of contacts 10–14 years of age in both sites. We observed another peak in mean number of contacts between persons 30–39 years of age and persons 40–59 years of age, driven mostly by conversation-only contacts (Appendix Figure, panels C, D). We noted few to no contacts reported for infants; however, the mean number of contacts between infants and other ages generally increased with age to peak at 10–14 years among rural residents and 30–39 years among urban dwellers.

#### Patterns of Contact by Location

In both urban and rural sites, the estimated number of co-located persons greatly exceeded the number of contacts reported by participants. Participants in the urban site reported a mean of 26.9 (95% CI 23.5–30.3) proximity contacts compared with 6.8 (6.5–7.1) close contacts; participants in the rural site reported a mean of 23.1 (20.3–25.9) proximity contacts and 9.8 (9.4–10.2) close contacts. The 3 locations with highest mean number of contacts were places of worship, schools, and transport hubs (Table 3). We found also that rural participants were more likely (n = 752 visits, 48%) to visit other homes compared with urban participants (n = 288 visits, 29%). Despite overall numbers being similar, the locations where contact occurred was meaningfully different between urban and rural sites.

**Table 3:** Number of times participants reported visiting a location and the median number of persons reported per location on day 1 in study of social contact patterns, Mozambique, 2021–2022*

Place visited	Rural			Urban	
	Reported visits to location, no. (%)	No. persons reported at location, median (IQR)		Reported visits to location, no. (%)	No. persons reported at location, median (IQR)
Other home	752 (48)	4 (3–6)		261 (29)	4 (3–7)
Street	369 (23)	2 (1–5)		342 (37)	4 (3–9)
Market/Shop	92 (6)	6 (3–12)		77 (8)	10 (3–30)
Transport/Hub	90 (5)	16 (11–18)		60 (6)	18 (12–20
Agricultural field	104 (7)	3 (1.8–6.3)		8 (1)	5.5 (1–6.8)
School	62 (3)	25 (15–25.8)		63 (6)	30 (24–33.5)
Work	45 (2)	6 (4–20)		54 (6)	6 (3–10)
Place of worship	26 (2)	20 (10.3–30)		23 (2)	30 (17.5–45)
Well	17 (1)	4 (2–6)		1	7
Playground	1	15		1	13
Other	45 (3)	10 (5–23)		47	7 (3.5–18)

*IQR, interquartile range.

#### Sensitivity of Transmission Model Disease Dynamics to Empirical Contact Matrices

**Figure 3:**
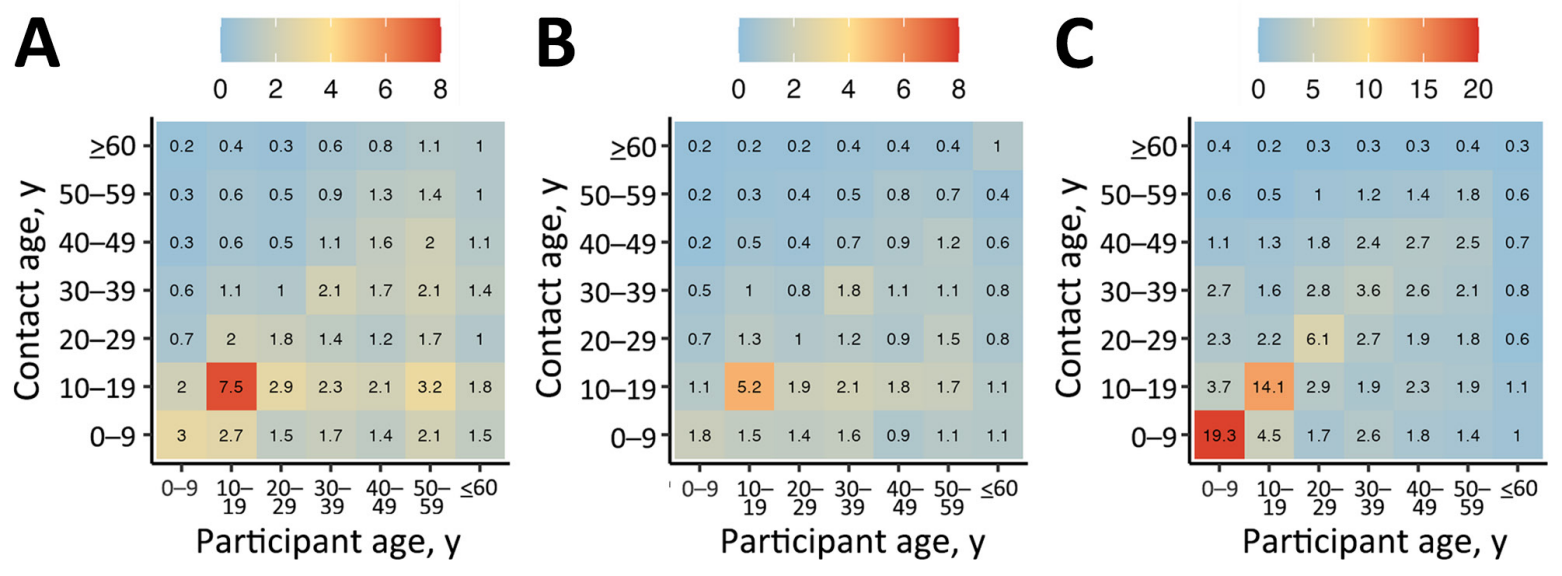
Contact matrices based on empiric data from study of human contact patterns Mozambique, 2022. A) Rural sites; B) urban site; C) synthetic contact matrix derived from Mozambique-specific demographic data by Prem et. al. (*12*).

To recap, we restructured the age-specific matrices (Figure 2) into 7 age groups (see Methods) (Figure 3, panels A, B). Assortative contacts in the empirical data were highest among those 10–19 years of age (higher in rural compared with urban) compared with synthetic values, which showed the highest number of assortative contacts for those 0–9 years of age (Figure 3, panel C) (*12*).

**Figure 4:**
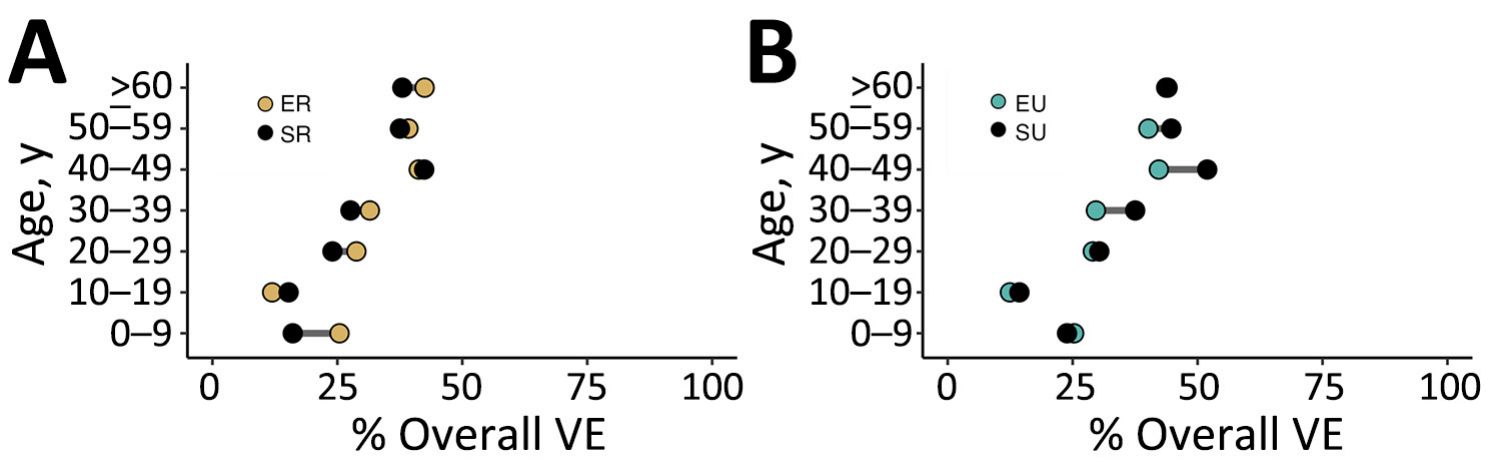
Modeled age-specific VE derived from study of human contact patterns, Mozambique, 2022. Overall VEs of a respiratory infection, comparing synthetic and empiric contact rates, are shown for rural (A) and urban (B) sites. ER, empiric rural; SR, synthetic rural; EU, empiric urban; SU, synthetic urban; VE, vaccine effectiveness.

The values of the empirical overall VE overlapped with synthetic values in most ages. However, synthetic VE values were marginally higher in the 10–19 years and 40–49 years age groups in the rural site (Figure 4, panel A). Data for the urban site showed synthetic VE values to be higher in all ages (particularly adults 30–59 years of age) except for children 0–9 years of age (Figure 4, panel B). Of note, our empirical data revealed higher attack rates in unvaccinated (AR) compared with vaccinated (ARv) persons (AR 94%, ARv 77%) and lower overall VE values (18%) for those 0–9 years of age compared with attack rates (rural AR 84%, rural ARv 62%, rural VEs 26%; urban AR 84%, urban ARv 62%, urban VEs 26%). On the basis of synthetic values, contacts among persons ≥60 years of age were underestimated compared with empirical values, producing notably lower attack rates among this age group (synthetic AR 49%, synthetic ARv 31%; rural AR 40%, rural ARv 24%; urban AR 36%, urban ARv 21%).

## Discussion

We present results from a 2-day cross-sectional study aiming to quantify social contact rates among residents of a rural and urban site in Mozambique during the COVID-19 pandemic. We engaged with the local community to get their views on the suitability and acceptability of our tools and study procedures. We made several key observations. First, we used the qualitative outcomes to modify the format and content of the paper diaries to make them more user friendly (Appendix). Second, participants from the rural site had significantly higher average number of contacts compared with the urban site. Third, the reported mean contacts increased with age to peak at school-going children and teenagers 15–19 years of age, and mean contacts were higher among adults (>18 years of age) compared with children <5 years of age. Fourth, mixing was assortative (increased frequency of contacts within the same age groups) among school-going children, with less pronounced intergenerational mixing, particularly in the urban site. Finally, in model simulations of a respiratory pathogen, we found meaningfully different attack rates and VE data among both child and elderly groups when comparing our local data with widely used contact matrices modeled from other settings.

In the early phases of the COVID-19 pandemic, Mozambique adopted physical distancing policies but no express requirement to stay at home (*23*). This was similar to measures implemented globally to reduce transmission of SARS-CoV-2. Only a few countries in sub-Saharan Africa—e.g., South Africa (*24*), Kenya (*3*,*5*), Zimbabwe (*4*), Uganda (*9*), and Somaliland (*8*)—had collected empirical social contact data from various settings before 2020. Reports from LMICs (e.g., Kenya, Malawi) regarding contact patterns during the pandemic remain sparse (*6*,*7*) compared with data reported from HICs (e.g., United Kingdom, Europe, United States) (*16*,*17*). Longitudinal social contact data during the pandemic, taking into account demographic, social, and economic contexts, would have been critical for a better understanding of the transmission pathways of the novel virus in LMICs. Such data might have provided insights into how to enhance nonpharmaceutical interventions and identify priority groups for immunization once vaccines became available (albeit in limited supply) in LMICs. In January 2021–April 2022, Mozambique experienced 3 waves of infection driven by the Beta (B.1.351), Delta (B.1.617.2), and Omicron SARS-CoV-2 variants (*19*,*25*). Because our study was designed to quantify precision by age rather than temporally, we were not able to fully quantify changes in the contact patterns over time and correlate this to the introduction and spread of the new variants of concern. We can speculate that contact patterns changed over time in response to initial spread followed by aggressive societal response followed by a complex, evolving situation of government and individual behavioral responses (*19*) as variants emerged (*25*), but given our study’s design, we cannot draw firm conclusions from the data we collected.

Compared with reported results from studies conducted during pandemic periods in Kenya (*7*) and Malawi (*6*), our data revealed lower mean number of contacts but a higher number of contacts reported by participants in the rural compared with the urban site. We interpret this information with care, however, because data from Kenya and Malawi were collected from high-density settlements, where persons may have been unable to fully adhere to physical distancing due to economic reasons. The government of Mozambique periodically revised physical distancing policies to curb the spread of SARS-CoV-2 (*26*), but we propose that those guidelines were not strictly followed, particularly by school-going children and working adults. Considering our data were collected from surveillance sites that were representative of the demographic distributions of the populations inhabiting them, we believe our data can be generalizable to similar areas in sub-Saharan Africa during the COVID-19 pandemic. Mozambique is the first of 4 countries that we surveyed as part of the GlobalMix Study (*18*). Results from the forthcoming sites will reveal the degree of heterogeneity among sites and, therefore, the generalizability.

We implemented several innovations in the GlobalMix Study. First, we collected contact data from participants over 2 consecutive days. We considered the negative potential of respondent fatigue and recall bias (leading to underreporting of contacts) relative to this investigation and undertook several measures to minimize these factors (Appendix). By iteratively asking the participants details of their contacts based on a self-reported, prepopulated list, we were able to prompt participants to remember most of their contacts, thereby potentially minimizing recall bias. Despite those efforts, reporting still decreased. However, the average number of contacts stratified across different covariates of interest remained relatively similar over 2 days, suggesting the stability of participants’ recall and of the number and nature of contacts made over multiple days. The stability of contact networks across days has been suggested in Kenya (*7*) and Malawi (*6*) through autonomous methods that minimize recall bias (*10*,*11*). Another innovation of our study was an estimation of group proximity contacts at locations frequently visited by participants. Of note, participants reported almost 4 times the number of proximity contacts compared with detailed individually reported contacts. This difference suggests the potential to substantially underestimate the number of interactions that could lead to transmission events, particularly in highly mobile age groups.

Finally, our transmission model simulation demonstrates the importance of contextual empirical social contact data. Although advanced methods for projecting social contact patterns onto regions without data exist (*12*), we found that age-specific infection attack rates from a model based on empirical contact data differed meaningfully compared with a model parameterized with synthetic contact rates. We found that the largest differences in attack rates (comparing vaccinated versus unvaccinated persons) resulted in increased VE in the youngest (0–9 years) age group, who often represent the most vulnerable group. These findings were consistent with a Uganda model, where use of local contact pattern data resulted in larger epidemics in young children and smaller epidemics in adults >35 years of age compared with using UK-based contact data (*9*). It is also notable that we observed distinct contact patterns resulting in divergent model results for the Mozambique rural and urban sites, which highlights the effect of subnational differences in contact patterns and its bearing on disease dynamics. Such insights are not possible with widely used, national-level contact data.

In conclusion, we present empirical results of a cross-sectional study quantifying rates and patterns of human social contacts relevant for the spread of directly transmitted infections in rural and urban sites in Mozambique. We demonstrated the possibility of collecting high-quality social contact data from resource-poor settings, reducing reliance on synthetic data modeled from HICs. We also demonstrated the potential advantages of empirical compared with synthetic data in a transmission and vaccine control model and advocate for the use of contextual data in similar studies. Questions remain regarding whether relaxing of nonpharmaceutical interventions might have influenced the social contact patterns in this setting. As the GlobalMix Study unfolds, we endeavor to make all our data collection tools, data, and analysis scripts findable, accessible, interoperable, and reusable. We hope that our continuing investigation efforts, which include completing data collection from 3 other LMICs, will provide greater insights into the techniques used in accessing human social contacts, thereby informing vaccine interventions.

Appendix Additional information for social contact patterns in rural and urban settings, Mozambique, 2021–2022.
