# Supplementary material for: Social Contact Patterns in Rural and Urban Settings, Mozambique, 2021–2022
Source: Emerg Infect Dis. 2025 Jan;31(1):94–103. doi: 10.3201/eid3101.240875 (PMC11682788; doi:10.3201/eid3101.240875)
Supplement: Appendix — Additional information for social contact patterns in rural and urban settings, Mozambique, 2021–2022. [file 24-0875-Techapp-s1.pdf]

Article DOI: http://doi.org/10.3201/eid3101.240875

EID cannot ensure accessibility for supplementary materials supplied by authors. Readers who have difficulty accessing supplementary content should contact the authors for assistance.

Social Contact Patterns in Rural and Urban Settings, Mozambique, 2021–2022

Appendix

Details of Fieldwork, Challenges Encountered, and Practical Solutions Applied

We introduced self-report paper diaries to the residents of the Manhiça and Polana Canhiço HDSS to record data on egocentric social contact patterns. It is important to note the logistical and technical challenges we experienced during the conduct of our study, and our mitigation measures, to better inform future studies in similar settings. Following extensive consultations with community members, we used paper diaries with contextualized questions and customized visual aids to guide age selection of contacts, including those met for the first time. During implementation, adult males were hard to find since most left their houses early for work, market sales and to tend to their farms. Absenteeism was also reported in a similar study in Kenya (*3*) and the authors replaced the loss-to-follow-up persons with others of similar demographic characteristics. Due to the COVID-19 pandemic and subsequent stay at home orders in Mozambique, both HDSS databases had not been updated and we resorted to door-to-door recruitment for under-5-year-olds and later expanding the study catchment area to additional HDSS blocks in the urban site. During the rainy season, remote areas in the rural site were inaccessible thus hampering recruitment and data collection exercises. Once recruited, some participants were unavailable to present their diary data during the exit visits, and almost half of diaries in both sites were partly filled in, thus requiring assistance from our field staff to complete the diary. Eventually, we resorted to recruiting children from mothers attending health clinics, visits to workplaces for adult males, and following up participants through phone calls. The lapse in time between diary data collection period and exit survey could have contributed to recall bias and it was not possible to ascertain the magnitude and direction of the recall bias.

Despite this, we had a good buy-in from the community due to our extensive community engagement conducted before and during the study, which also resulted in higher participation rates in general than previously observed in other sub-Saharan African settings. Furthermore, the data was collected during periods of active transmission of SARS-CoV-2 in the community, including certain times when certain restrictive measures were in place, such as the closure of schools and parks, limitations on the number of employees at the workplace and passengers on public transportation, social distancing, and other measures (*23*). This context likely caused some disruption, given the contingency scenario at the time, and may have influenced the characteristics of social contact patterns due to the ongoing pandemic. Additional measures that we took sequentially to minimize recall bias and respondent fatigue are:

a) During enrollment, the field team listed all household members for each participant in the paper diary and electronic diary designed in REDCap.

b) During diary collection, the list of household members was generated automatically in the electronic diary. The field team marked as "yes/no" whether the participant had a contact with each of the household members listed. Then, we listed all nonhousehold members separately.

c) For Day 2 diary, the list of household and nonhousehold members was again populated automatically and each contact marked as "yes/no" as above. Then, we listed any additional contacts not captured in the 2 lists above.

Appendix Table. Median and interquartile ranges for rural and urban contacts on Day 2 in a study of social contact patterns in rural and urban settings, Mozambique, 2021–2022*

Characteristic	Rural, median (IQR)	Urban, median (IQR)
Sex		
F	5 (3–9)	5 (3–8)
M	8 (5–11)	6 (4–8)
Age		
<6 mo	5 (3–7)	2 (1–4)
6–11 mo	5 (3–9)	3.5 (2–5.8)
1–4 y	5 (3–8)	5 (3–7)
5–9 y	6 (4–9)	4 (2–5)
10–14 y	6 (3–9)	4 (3–7)
15–19 y	7 (4–12)	6 (3–7)
20–29 y	6 (4–9)	4 (3–5)
30–39 y	5 (3–10)	4 (2.8–6)
40–59 y	5 (3–9)	3 (2–4)
≥60 y	4 (2–7)	3 (1.3–4.8)
Occupation/daily activity		
Child	5 (3–8)	3 (2–5)
Unemployed	4.5 (2–8)	3.5 (2–5.3)
Student	6 (4–10)	4 (3–7)
Homemaker	3 (2–6.5)	2 (2–3)
Casual laborer	6.5 (2.8–10.3)	3.5 (2–4.8)
Farmer	5 (2–7)	9 (5–9)
Businessperson	9 (4–9.5)	3 (2–4)
Office worker	5 (3.5–11)	4 (2–5)
Retired	7 (5.8–7)	1.5 (1–2.8)
Other	5.5 (4–10)	5 (4–6)
Household size		
1	6 (2–10)	5.5 (4–8)
2–3	5.5 (3–9)	4 (2–6)
4–6	5 (3–8.8)	4 (2–5)
7–10	5 (3–7.8)	3 (2–5)
≥10	5 (3–6.8)	2 (2–5)
Enrolled in school		
Y	6 (3–10)	4 (2–7)
N	5 (3–9)	3 (2–5)
Weekday/Weekend		
Weekday	5 (3–9)	4 (2–6)
Weekend	5 (3–9)	4 (2–5)
ARI symptoms		
No symptom	5 (3–9)	4 (2–6)
>1 symptom	6 (4–9)	4 (3–6)
AGE symptoms		
Y	7 (3–12)	3 (2–5)
N	5 (3–9)	4 (2–6)

*AGE, acute gastroenteritis; ARI, acute respiratory infection, IQR, interquartile.

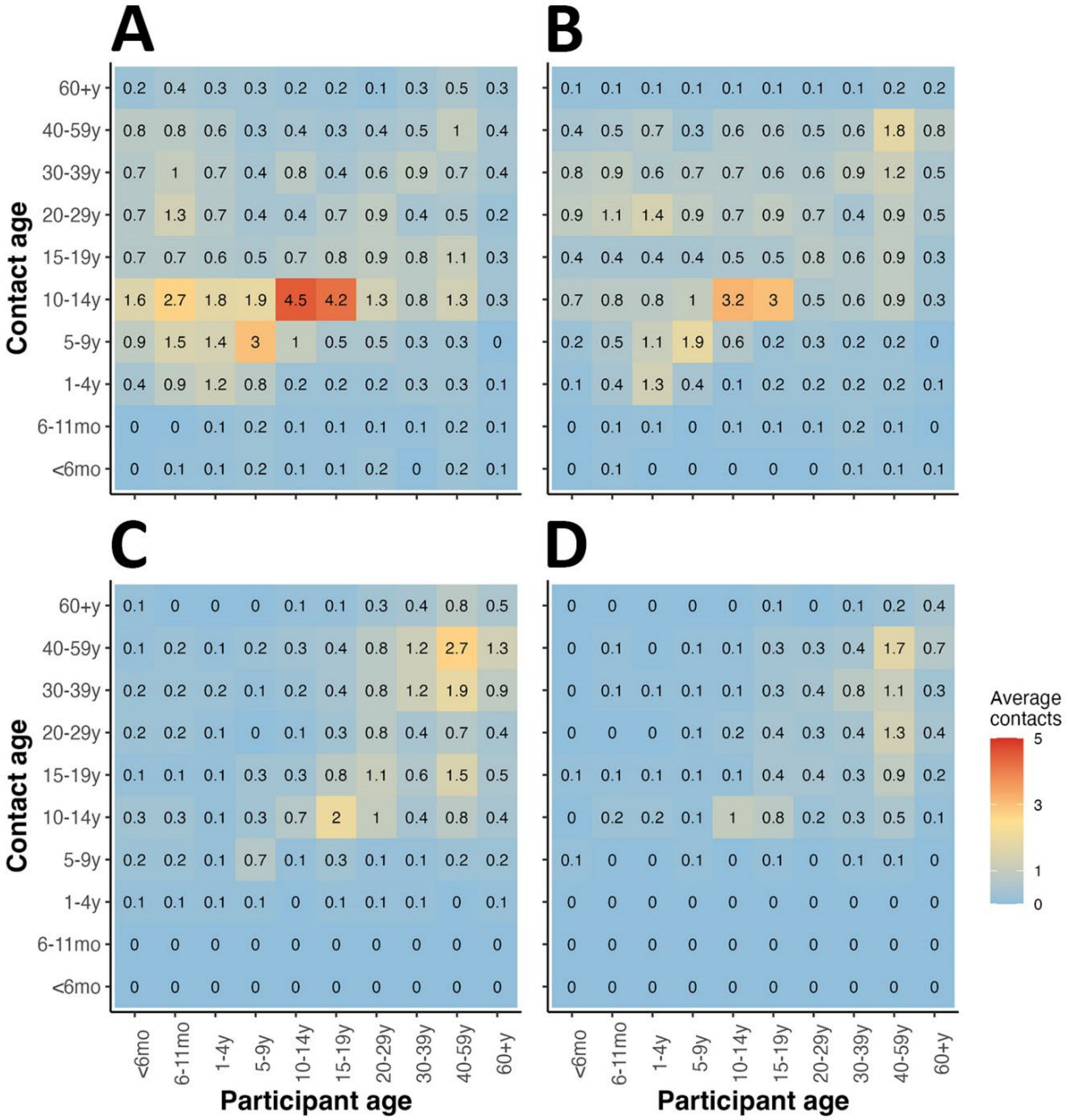

Appendix Figure. Contact matrices stratified by type of contact (physical or conversation) in rural and urban sites in Mozambique, 2021–2022. Panels A and B show social contacts that involved touch (physical contacts) in rural (A) and urban (B) sites. Panels C and D show conversation-only contacts in rural (C) and urban (D) sites.
